# Supplementary figures and images for: Caspase-1 activates gasdermin A in non-mammals
Source: eLife. 2024 Mar 18;12:RP92362. doi: 10.7554/eLife.92362 (PMC10948149; doi:10.7554/eLife.92362)

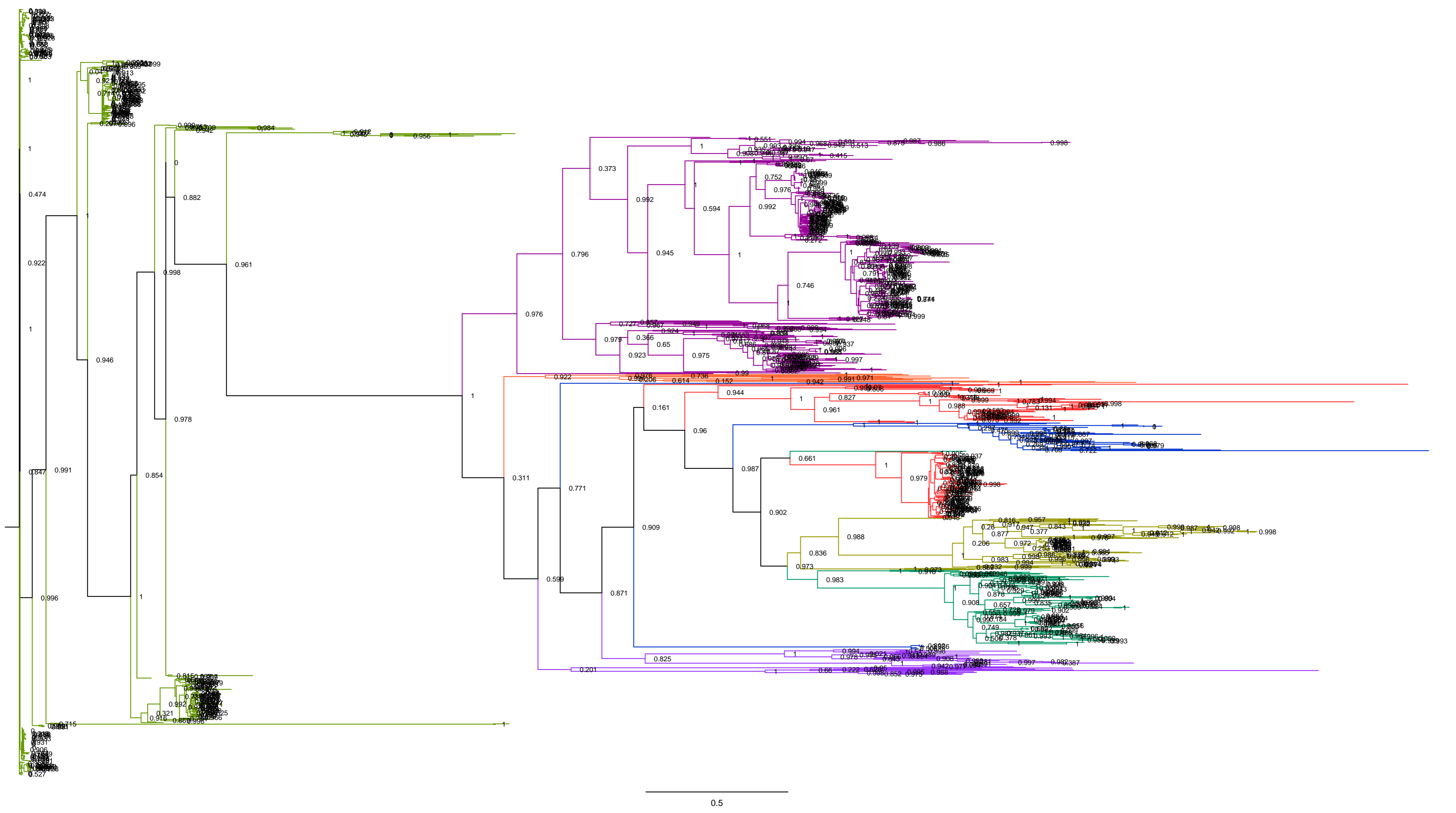

Supplement: Figure 1—figure supplement 3—source data 2. [file elife-92362-fig1-figsupp3-data2.pdf]

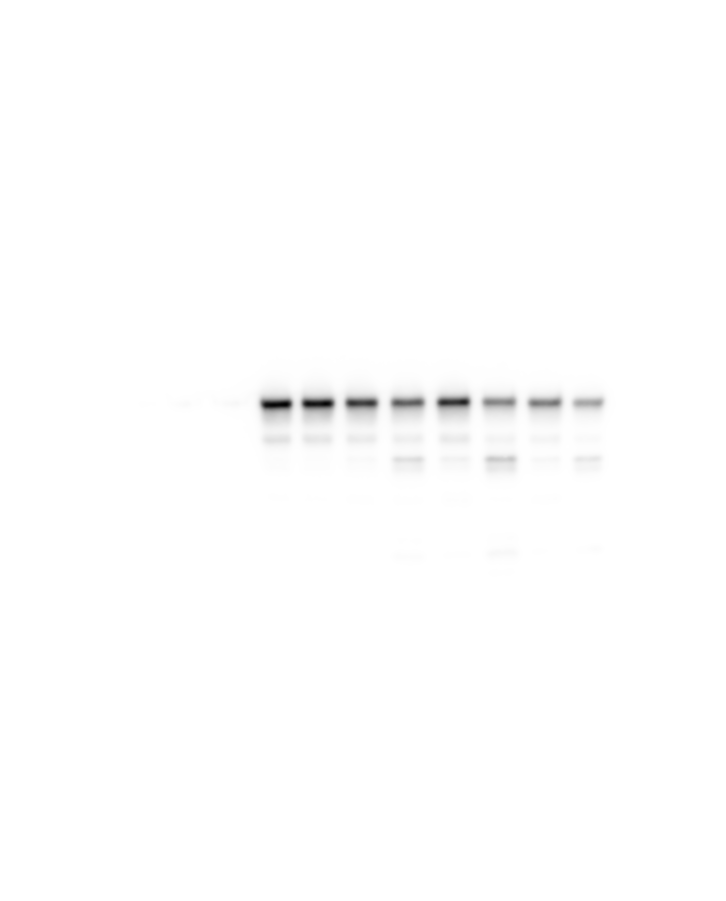

Supplement: Figure 3—source data 2. [file elife-92362-fig3-data2.zip › Figure 3source data 2.tif]

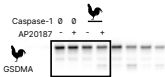

Supplement: Figure 3—source data 3. [file elife-92362-fig3-data3.pdf]

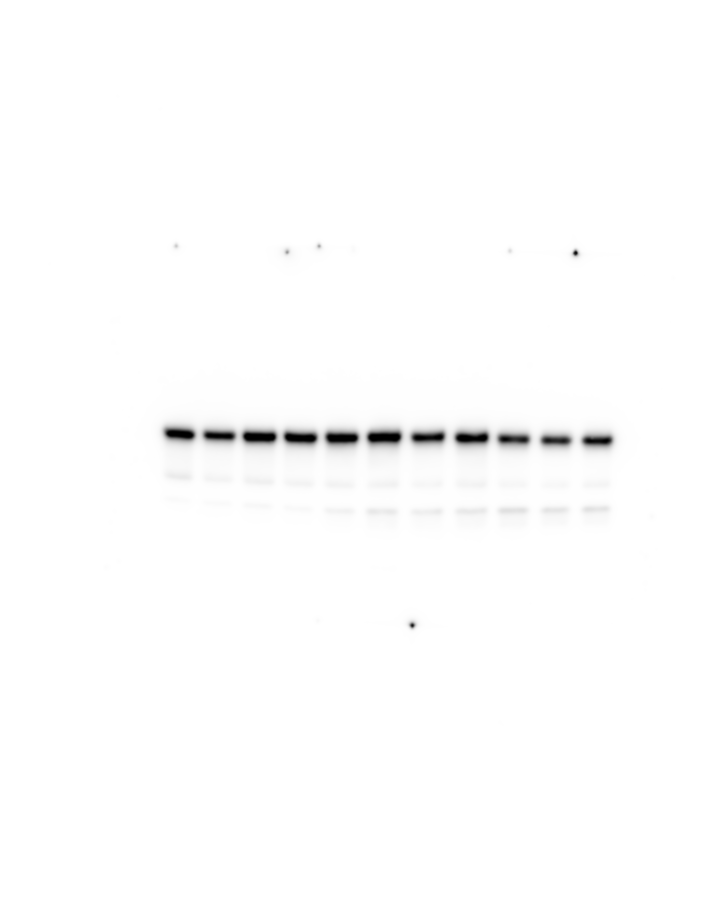

Supplement: Figure 3—source data 4. [file elife-92362-fig3-data4.zip › Figure 3source data 4.tif]

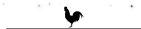

⌚ Time

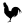  
GSDMA

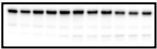

Supplement: Figure 3—source data 5. [file elife-92362-fig3-data5.pdf]

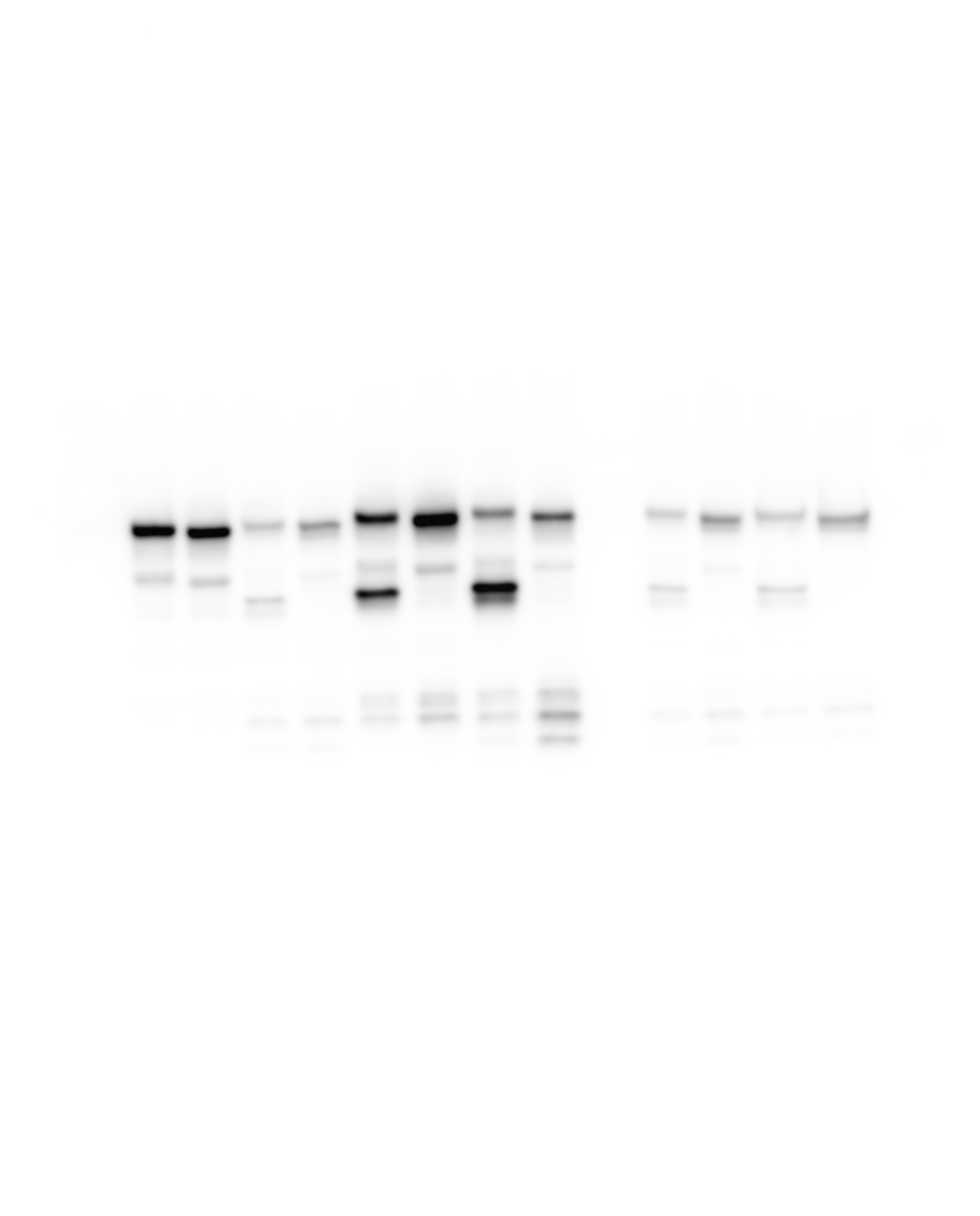

Supplement: Figure 3—source data 6. [file elife-92362-fig3-data6.zip › Figure 3source data 6.tif]

Caspase-1     $\emptyset$     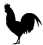    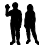    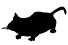

P1    D    A    D    A    D    A    D    A

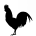  
GSDMA

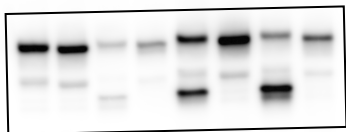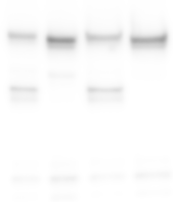

Supplement: Figure 3—source data 7. [file elife-92362-fig3-data7.pdf]

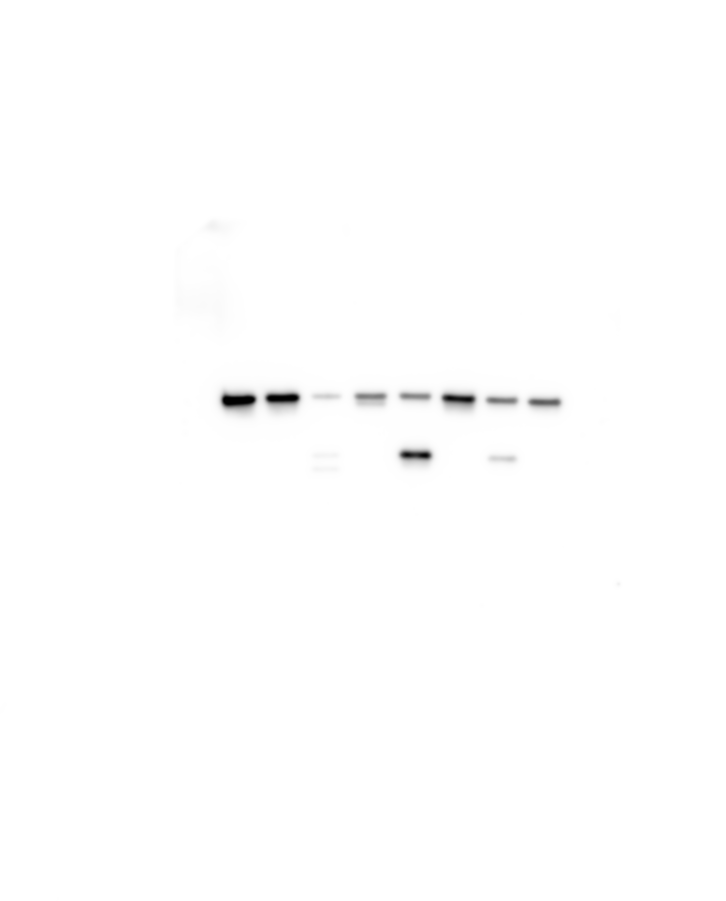

Supplement: Figure 3—source data 8. [file elife-92362-fig3-data8.zip › Figure 3source data 8.tif]

Caspase-1

0

P1

D

A

D

A

D

A

D

A

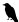

GSDMA

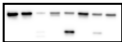

Supplement: Figure 3—source data 9. [file elife-92362-fig3-data9.pdf]

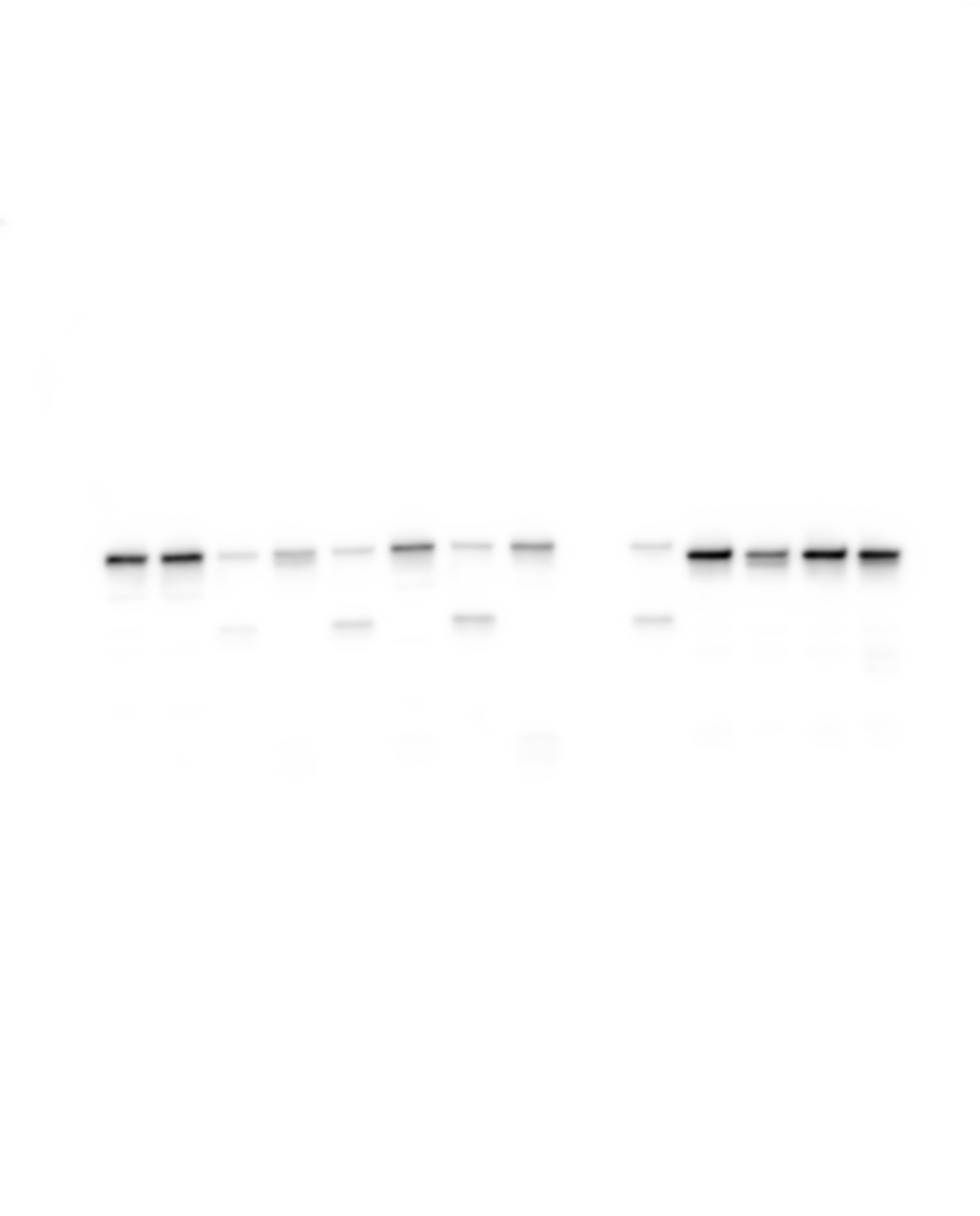

Supplement: Figure 3—source data 10. [file elife-92362-fig3-data10.zip › Figure 3source data 10.tif]

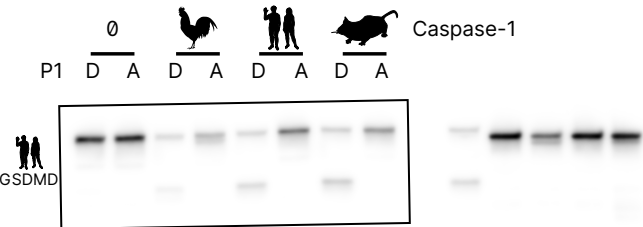

Supplement: Figure 3—source data 11. [file elife-92362-fig3-data11.pdf]

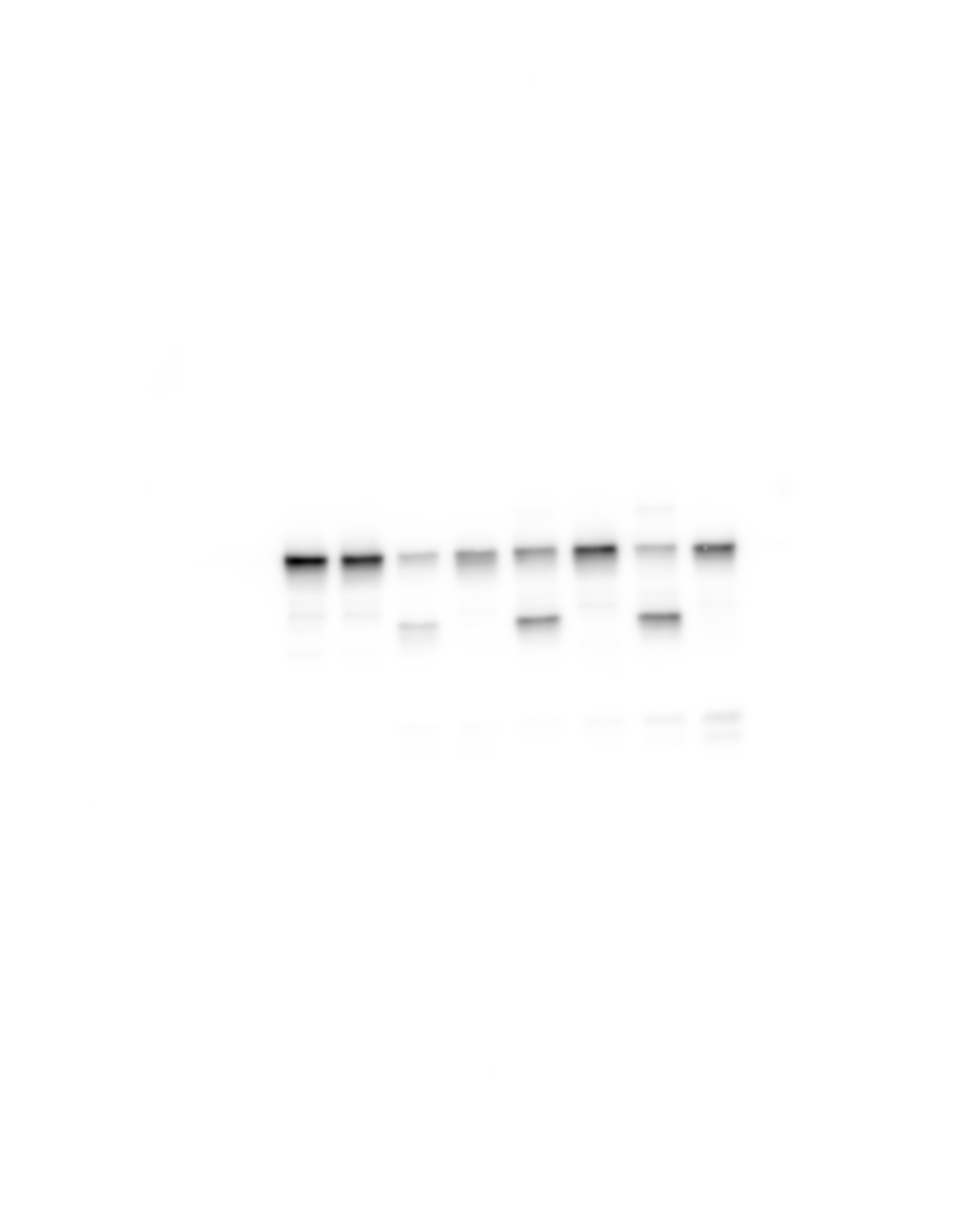

Supplement: Figure 3—source data 12. [file elife-92362-fig3-data12.zip › Figure 3source data 12.tif]

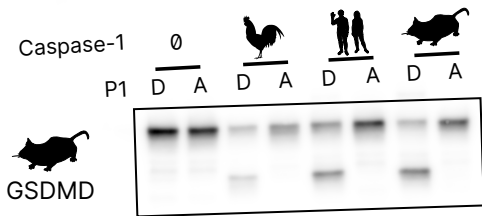

Supplement: Figure 3—source data 13. [file elife-92362-fig3-data13.pdf]

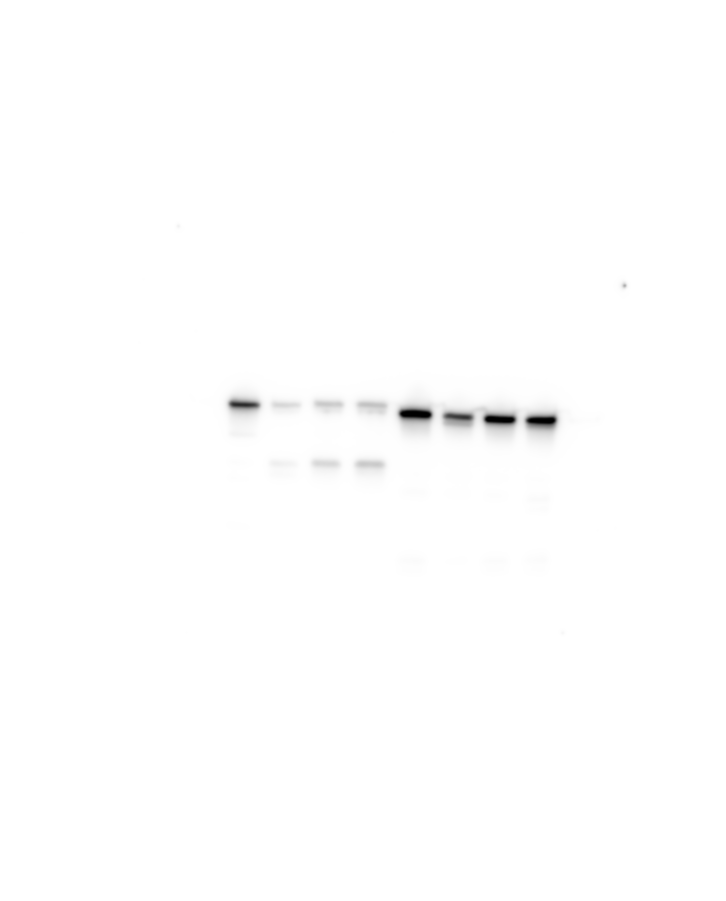

Supplement: Figure 3—source data 14. [file elife-92362-fig3-data14.zip › Figure 3source data 14.tif]

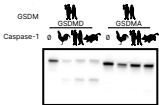

Supplement: Figure 3—source data 15. [file elife-92362-fig3-data15.pdf]

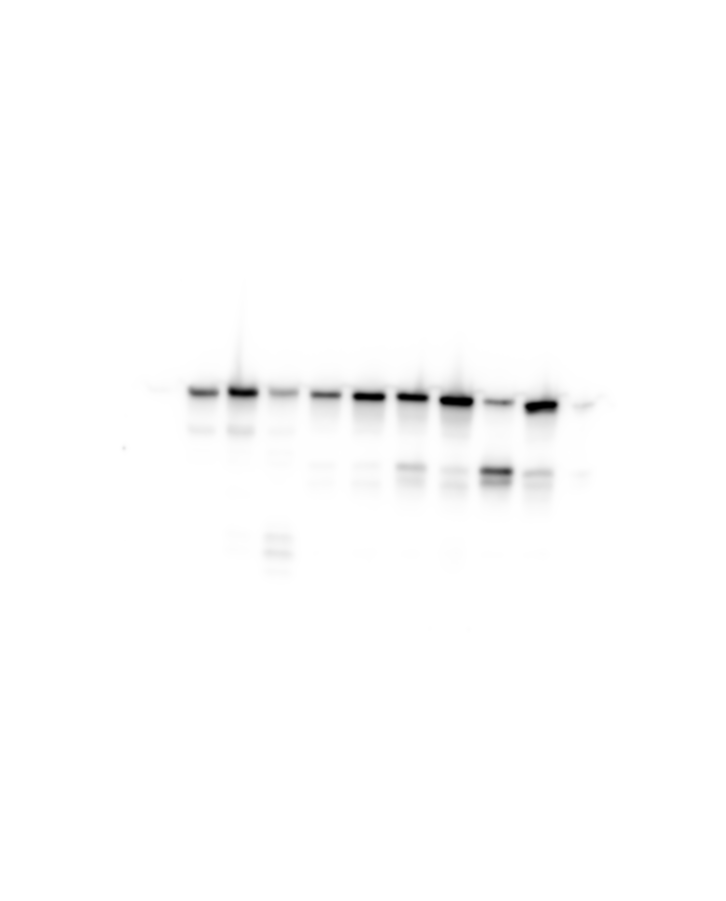

Supplement: Figure 3—source data 16. [file elife-92362-fig3-data16.zip › Figure 3source data 16.tif]

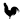

GSDMA

|           |   |   |   |
|-----------|---|---|---|
| Caspase-9 | - | + | + |
| AP20187   | - | - | + |
| P1        | D | D | D |

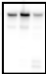

Supplement: Figure 3—source data 17. [file elife-92362-fig3-data17.pdf]

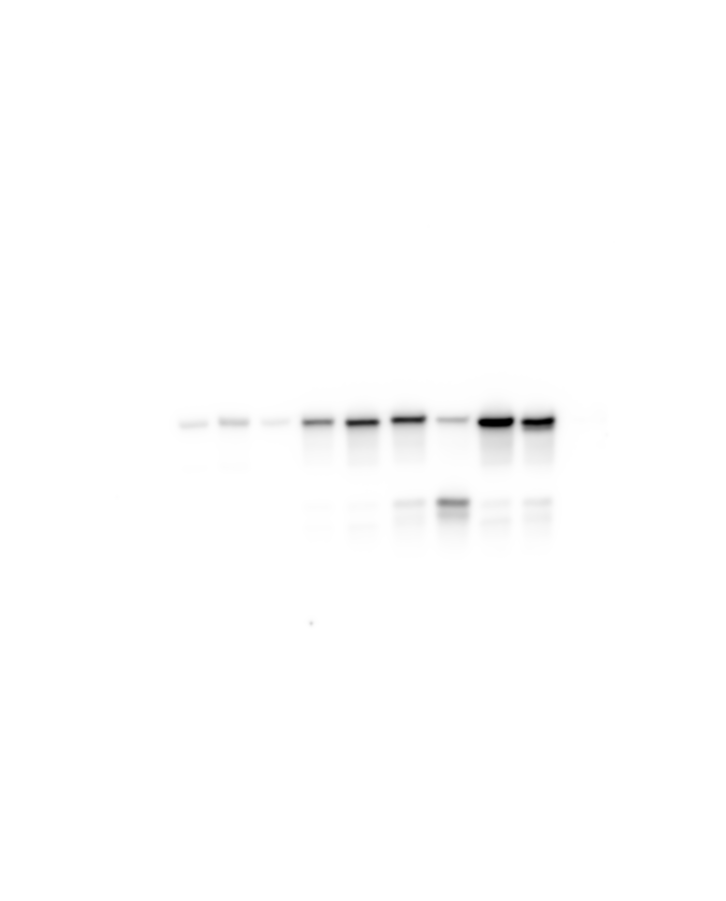

Supplement: Figure 3—source data 18. [file elife-92362-fig3-data18.zip › Figure 3source data 18.tif]

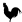

GSDME

Caspase-9  
AP20187  
P1

|   |   |   |   |   |   |
|---|---|---|---|---|---|
| - | - | + | + | + | + |
| - | - | - | + | - | + |
| D | A | D | D | A | A |

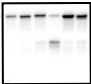

Supplement: Figure 3—source data 19. [file elife-92362-fig3-data19.pdf]

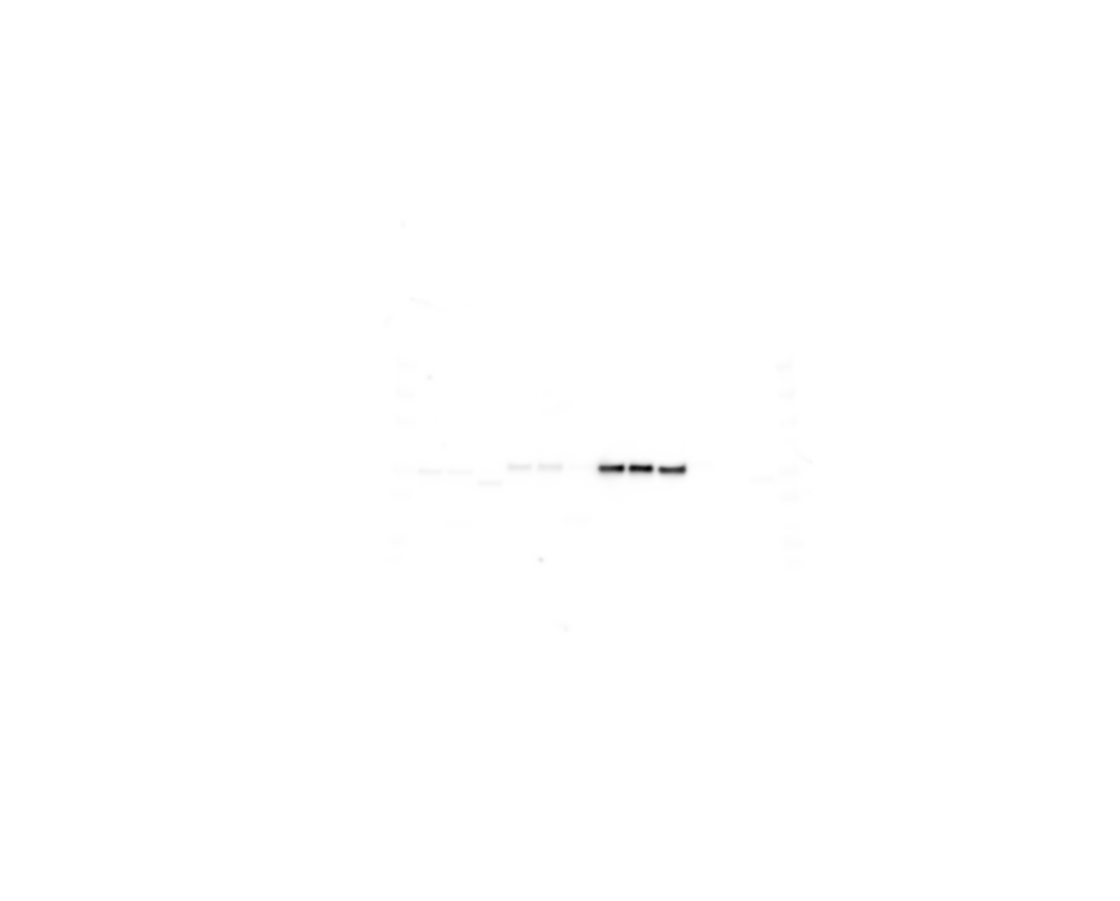

Supplement: Figure 3—figure supplement 2—source data 1. [file elife-92362-fig3-figsupp2-data1.zip › Figure 3figure supplement 2source data 1.tif]

|            | Crow<br>GSDMA |   |   | Crow<br>GSDME |   |   | Human<br>GSDMA |   |   | Human<br>GSDMD |   |   |
|------------|---------------|---|---|---------------|---|---|----------------|---|---|----------------|---|---|
| Human CASP | 0             | 1 | 3 | 0             | 1 | 3 | 0              | 1 | 3 | 0              | 1 | 3 |

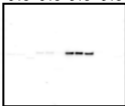

Supplement: Figure 3—figure supplement 2—source data 2. [file elife-92362-fig3-figsupp2-data2.pdf]

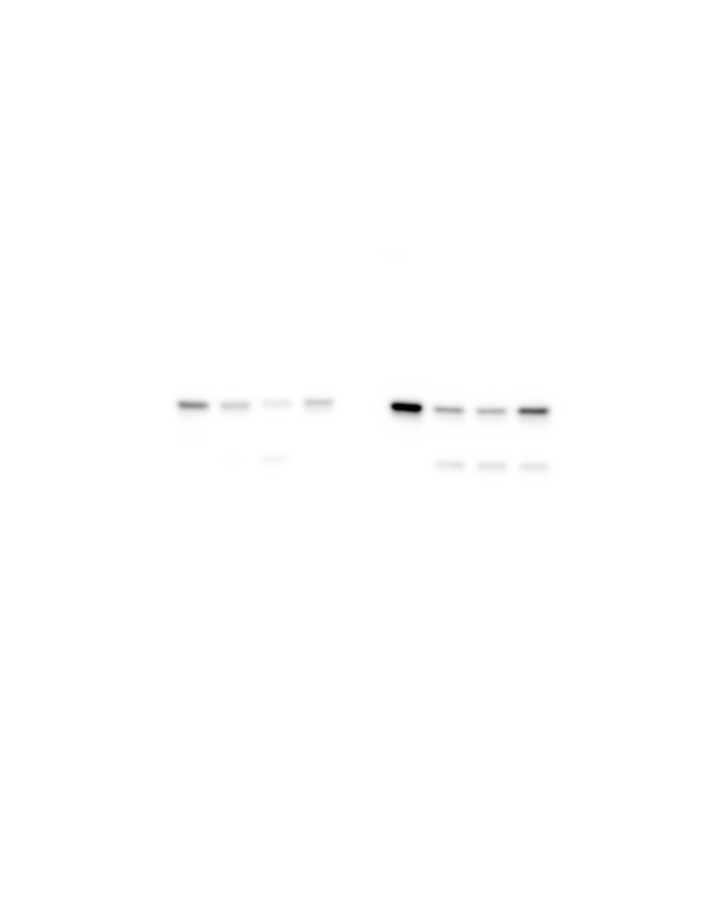

Supplement: Figure 5—source data 4. [file elife-92362-fig5-data4.zip › Figure 5source data 4.tif]

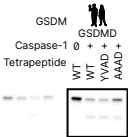

Supplement: Figure 5—source data 5. [file elife-92362-fig5-data5.pdf]

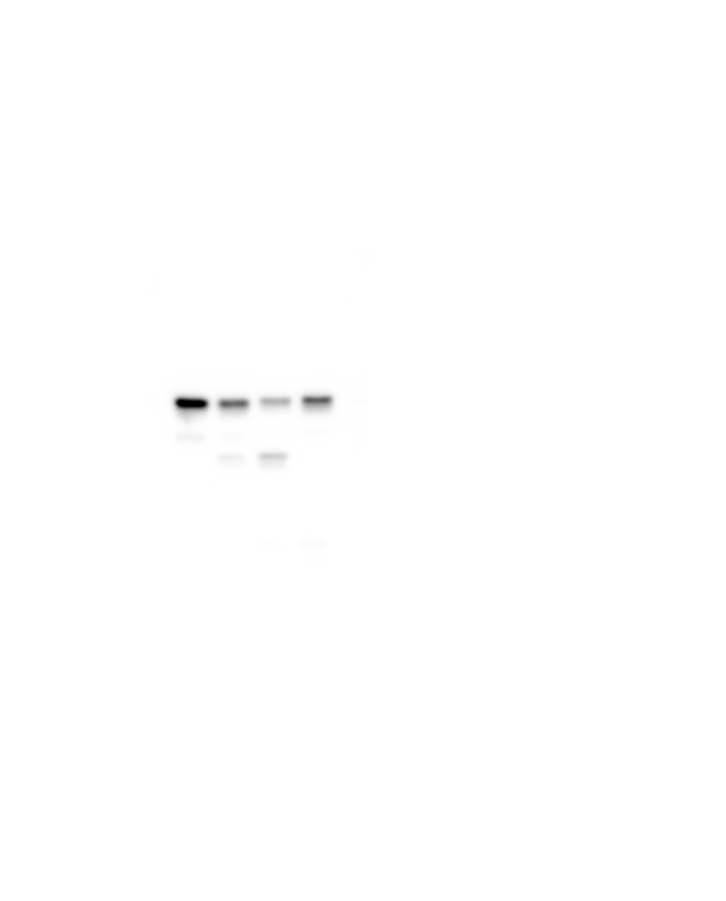

Supplement: Figure 5—source data 6. [file elife-92362-fig5-data6.zip › Figure 5source data 6.tif]

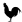

GSDMA

|              |    |    |      |      |
|--------------|----|----|------|------|
|              | 0  | +  | +    | +    |
| Caspase-1    |    |    |      |      |
| Tetrapeptide | WT | WT | YVAD | AAAD |

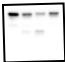

Supplement: Figure 5—source data 7. [file elife-92362-fig5-data7.pdf]

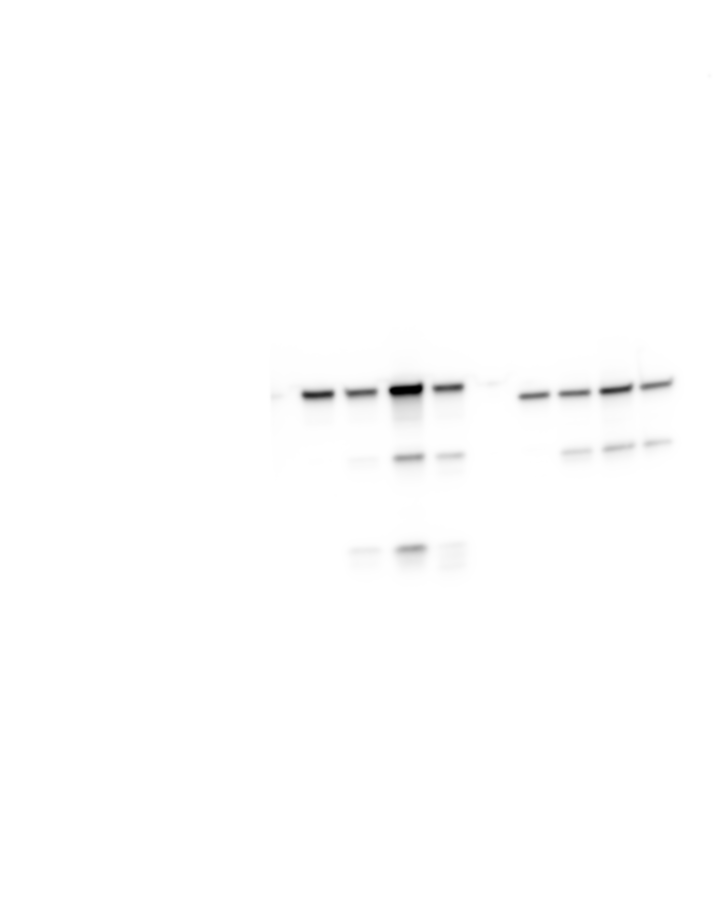

Supplement: Figure 6—source data 1. [file elife-92362-fig6-data1.zip › Figure 6source data 1.tif]

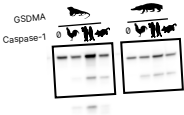

Supplement: Figure 6—source data 2. [file elife-92362-fig6-data2.pdf]

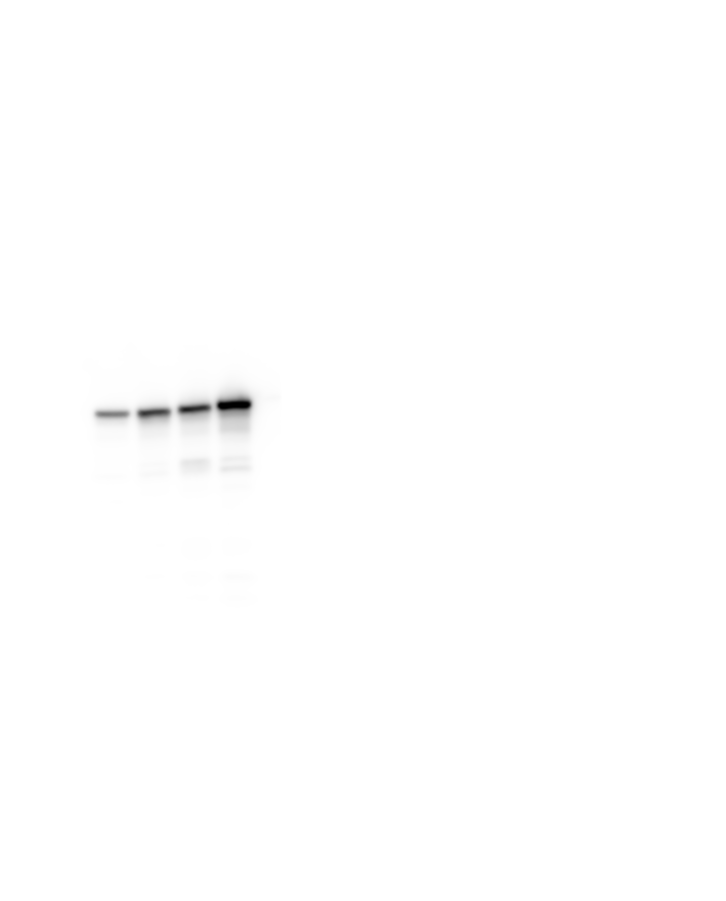

Supplement: Figure 6—source data 3. [file elife-92362-fig6-data3.zip › Figure 6source data 3.tif]

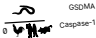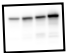

Supplement: Figure 6—source data 4. [file elife-92362-fig6-data4.pdf]

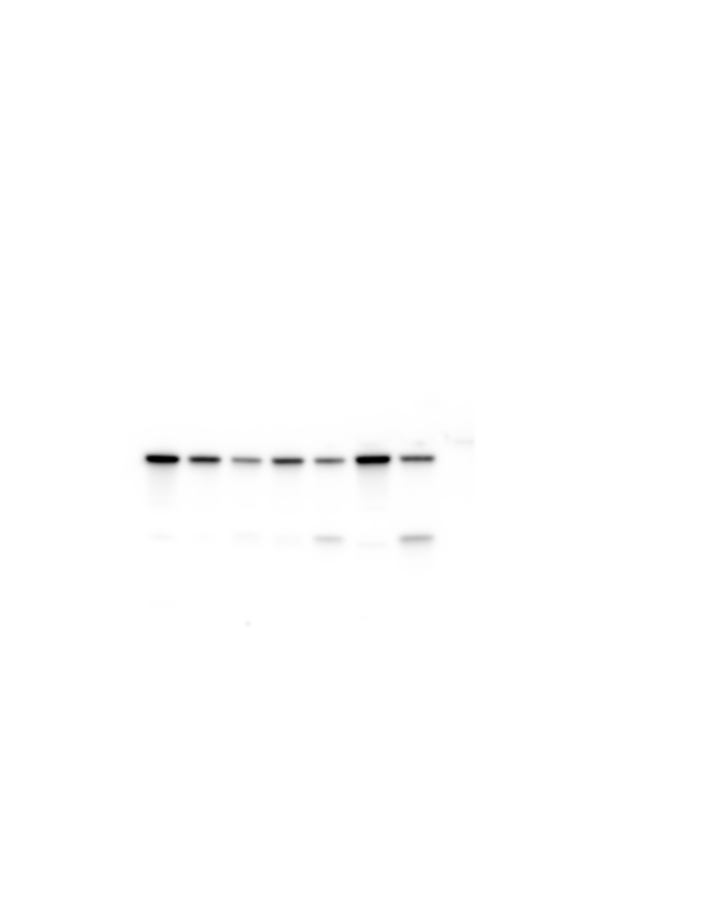

Supplement: Figure 6—source data 5. [file elife-92362-fig6-data5.zip › Figure 6source data 5.tif]

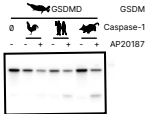

Supplement: Figure 6—source data 6. [file elife-92362-fig6-data6.pdf]

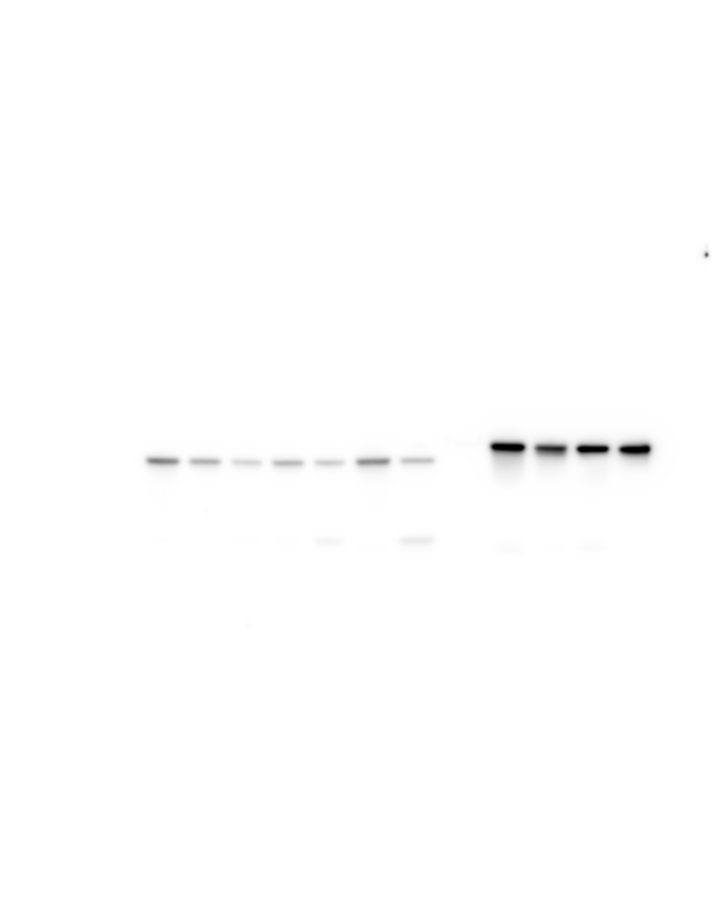

Supplement: Figure 6—source data 7. [file elife-92362-fig6-data7.zip › Figure 6source data 7.tif]

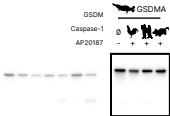

Supplement: Figure 6—source data 8. [file elife-92362-fig6-data8.pdf]

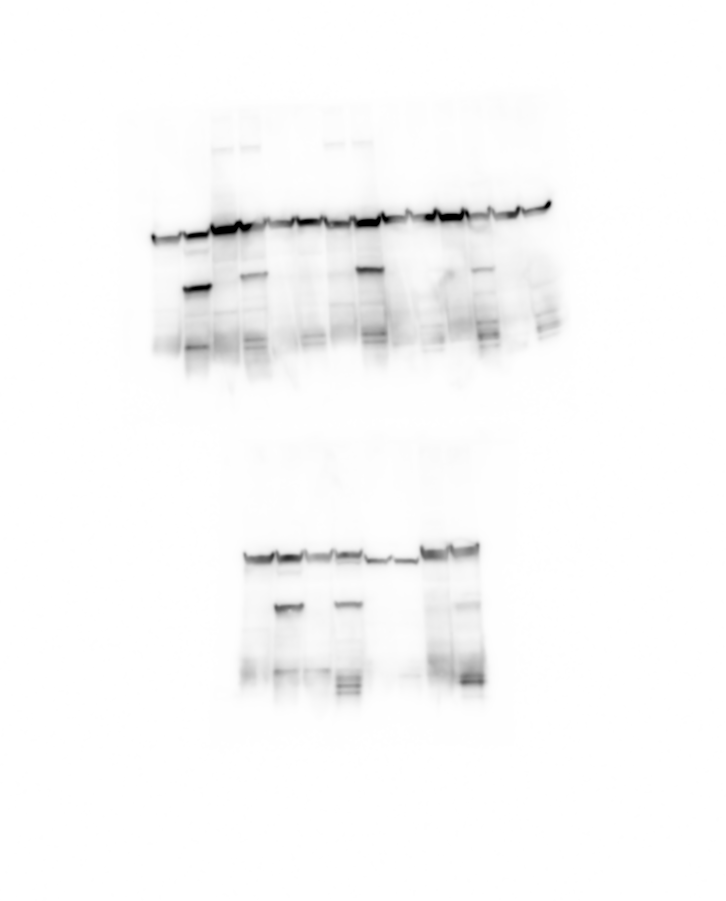

Supplement: Figure 6—source data 9. [file elife-92362-fig6-data9.zip › Figure 6source data 9.tif]
