# Supplementary material for: Caspase-1 activates gasdermin A in non-mammals
Source: eLife. 2024 Mar 18;12:RP92362. doi: 10.7554/eLife.92362 (PMC10948149; doi:10.7554/eLife.92362)
Supplement: Figure 6—source data 10. [file elife-92362-fig6-data10.pdf]

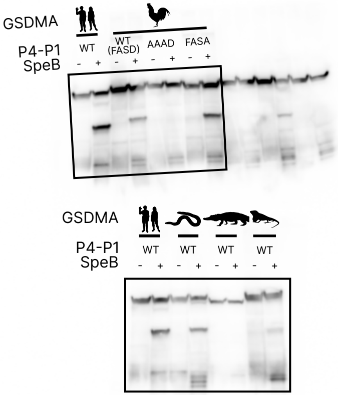
GSDMA
P4-P1
SpeB
WT
WT (FASD)
AAAD
FASA
- + - + - + - +
GSDMA
P4-P1
SpeB
WT
WT
WT
WT
- + - + - + - +
