# Supplementary material for: Caspase-1 activates gasdermin A in non-mammals
Source: eLife. 2024 Mar 18;12:RP92362. doi: 10.7554/eLife.92362 (PMC10948149; doi:10.7554/eLife.92362)

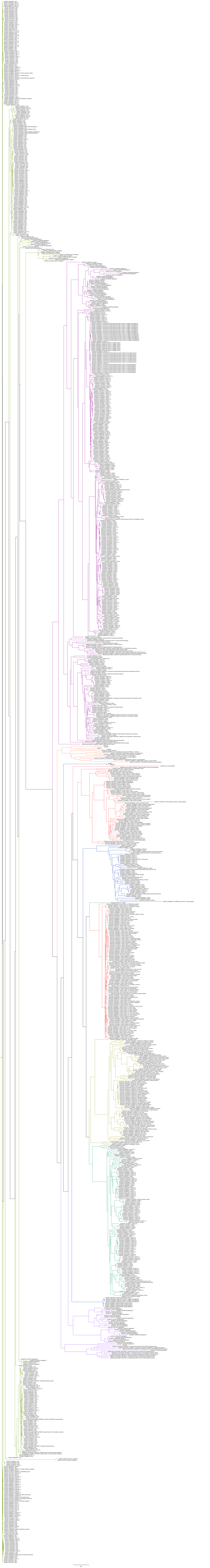

Supplement: Figure 1—figure supplement 3—source data 3. [file elife-92362-fig1-figsupp3-data3.pdf]
